# The Influence of Gestational Diabetes Mellitus upon the Selected Parameters of the Maternal and Fetal System of Insulin-Like Growth Factors (IGF-1, IGF-2, IGFBP1-3)—A Review and a Clinical Study

**DOI:** 10.3390/jcm9103256

**Published:** 2020-10-12

**Authors:** Tomasz Gęca, Anna Kwaśniewska

**Affiliations:** Chair and Department of Obstetrics and Pathology of Pregnancy, Medical University of Lublin, Staszica 16 street, 20-081 Lublin, Poland; haniakwasniewska@gmail.com

**Keywords:** insulin like-binding proteins, gestational diabetes mellitus, insulin-like growth factor I, insulin-like growth factor II

## Abstract

Background: Gestational diabetes mellitus (GDM), defined as impaired glucose tolerance with onset or first recognition in pregnancy, increases the risk of not only maternal but also fetal and neonatal complications. Given the structural similarity of insulin-like growth factors with insulin and participation of components of the insulin-like growth factor system in glucose homeostasis, we hypothesized that the IGF axis is involved in the development of GDM complications or its pathogenesis. The aim of this study was to evaluate the effect of GDM on the selected parameters of the insulin-like growth factors (IGF-1, IGF-2, IGFBP1-3) in the maternal and fetal blood. Methods: The clinical material of this case-control study included 109 pregnant women and their offspring. The study group (*n* = 120) consisted of 60 patients with diagnosed gestational diabetes and their newborn babies. The control group (*n* = 98) comprised 49 healthy parturients and their offspring. We measured the concentrations of IGF-1, IGF-2, IGFBP-1, IGFBP-2, IGFBP-3, insulin and glucose made by the ELISA method in peripheral blood serum in patients suffering from GDM and pregnant women without GDM, and in the umbilical cord blood of newborn babies born to them. Results: The analysis of concentrations of IGF-1, -2 and IGFBP-3 in peripheral blood as well as umbilical cord blood did not demonstrate a statistically significant difference between the study group and the control group. Significantly lower concentration of IGFBP-1, IGFBP-2 in peripheral blood and in umbilical cord blood was detected in the study group in comparison to the control group. A statistically positive correlation between the concentration of IGF-1 in umbilical cord serum of newborn babies born to women with gestational diabetes and the length of a baby after its birth was observed. Conclusions: Gestational diabetes mellitus does not significantly affect the concentrations of IGF-1, -2, IGFBP-3 in the peripheral blood and umbilical cord blood, but has the greatest influence on maternal and fetal IGFBP-2 concentrations. A positive correlation between the concentration of IGF-1 in umbilical cord blood and the length of a newborn suggests an influence of IGF-1 on the process of fetal development.

## 1. Introduction

Gestational diabetes mellitus (GDM) is defined as any impaired glucose tolerance, which appeared for the first time during pregnancy or was then identified [1]. Due to the expected increase in the prevalence of GDM, it seems crucial to understand the pathogenesis of the disease itself, as well as the related complications [2].

The system of insulin-like growth factors consists of two ligands IGF-1 and IGF-2 (insulin-like growth factor 1 and 2), two receptors IGFR-1, IGFR-2 (insulin-like growth factor receptor 1 and 2), six IGF binding proteins—IGFBP1-6 (insulin-like growth factor binding proteins 1-6) and four insulin-like growth factor binding protein-related peptides IGFBP-Rp1-4. The individual components of this system are characterized by a wide range of metabolic and mitogenic activities. The structural similarity of IGF-1 to insulin and the proven hypoglycemic effect regulated by IGFBP points to the participation of various components of this system in the regulation of glucose metabolism [3]. On the basis of the research conducted so far, we may conclude that changes in the system of insulin-like growth factors in women with gestational diabetes mellitus exert a potential role, not only in the development of the disease itself, but also in certain complications, for example, in fetal macrosomia [4,5]. The studies conducted so far on insulin-like growth factors in gestational diabetes are inconsistent and focus on the assessment of IGF in either the maternal or fetal compartment [6,7].

The aim of this study was to evaluate the effect of GDM on the insulin-like growth factors (IGF-1, IGF-2) and insulin-like growth factor binding proteins (IGFBP 1-3) in the maternal and fetal blood.

## 2. Materials and Methods

The clinical material of this work included 109 women who were consecutively admitted into the Clinic of Obstetrics and Pregnancy Pathology of the Independent Public Clinical Hospital No. 1 in Lublin from 2016–2017 to deliver a baby. The study group comprised 60 patients with diagnosed GDM, as well as 60 newborns delivered by them. The control group consisted of 49 healthy parturients without coexisting diseases and their offspring (49 new-born children). All the patients, after reviewing the purpose and means of conducting the research, gave informed consent in writing for a participation in this project. The consent to carry out the research was released by the Bioethical Commission at the Medical University in Lublin (resolution number KE-0254/210/2016). The patients with diagnosed GDM were divided into two subgroups, depending on the treatment applied. In the treatment of women with GDM G1, a diabetic diet was used, also taking into consideration moderate physical effort adjusted to an advanced state of pregnancy and the pregnancy course. Whereas in the subgroup of patients with GDM G2, apart from a diabetic diet and regular physical exercise, insulin therapy was also used. GDM was diagnosed on the basis of the criteria of WHO adopted in the year 2013: “Diagnostic Criteria and Classification of Hyperglycaemia First Detected in Pregnancy” [1].

The examination excluded patients who, on the basis of a clinical examination and the results of additional tests, were diagnosed with: chronic arterial hypertension, gestational hypertension, pre-eclampsia, eclampsia, liver diseases, endocrine disorders and autoimmune diseases, in order to avoid the impact of these diseases on the test results.

For the sake of the research, authors used samples of serum separated from peripheral blood (basilic vein), of women with at least 8 h since the last meal, at the beginning of the first period of labor, as well as umbilical cord blood taken immediately after the delivery of a baby (before delivering placenta) from the umbilical artery. The blood was sampled into disposable tubes S-Monovette type, 9 mm in volume (vacuum-aspiraton kits manufactured by Sarstedt, Germany), containing the blood clotting activator. In order to form a clot, the samples were left in an upright position for at least 30 min at room temperature. Next, they were spinned in a centrifuge (Sigma 1-6P, Polygen) for 10 min at room temperature, at the speed of 3800 rpm. The separation of serum took place under 1 h of taking the blood. The serum, obtained in such a way, was portioned in 200 μL per tube (0.5 mL), Eppendorf type (Medlab Products) and stored in a low-temperature freezer at −75 °C (Platinum Angelantoni 500, Italy) up to a year without several freezing or thawing, until the determination of concentrations.

The concentrations of IGF-1, IGF-2, IGFBP-1, IGFBP-2, IGFBP-3, insulin and glucose made by the ELISA method (Enzyme-linked Immunosorbent Assay) were measured in peripheral blood serum in the group of patients with GDM and in healthy patients, as well as in the umbilical cord blood serum of newborns born to them. Marking was made with the use of commercially available tests: IGF-1-ELISA (Enzyme Immunoassay for Quantitative Determination of human Insulin-like Growth Factor-I, Mediagnost, Germany), IGF-2-ELISA (Enzyme Immunoassay for Quantitative Determination of Human Insulin-like Growth Factor-II, Mediagnost, Germany), IGFBP-1-ELISA (Enzyme Immunoassay for quantitative Determination of human Insulin-like Growth Factor Binding Protein-1, Demeditec Diagnostics GmbH, Germany), IGFBP-2-ELISA (Enzyme Immunoassay for quantitative Determination of Insulin-like Growth Factor Binding Protein -2, Mediagnost, Germany), IGFBP-3-ELISA (Enzyme Immunoassay for Quantitative Determination of human Insulin-like Growth Factor Binding Protein -3, Mediagnost, Germany). Measurement of the absorbance was made at a wavelength of 450 nm, using a BIO-RAD microplate ELISA reader (Microplate Leader, Model 680, USA). The computer coupled with the reader by means of the Microplate Manager Version 5.2.1 program (BIO-RAD Laboratories, USA), based on the readings of light absorbance from the wells with known concentrations of standards, automatically drew standard curves on the basis of which it calculated the proteins concentration in the tested samples. All samples were assayed in triplicate. CV was <10%. Then, the insulin resistance indicator HOMA-IR (Homeostasis Model for Assessment of Insulin Resistance) was calculated, according to the following formula: HOMA-IR = glucose concentration [mmol/L] × insulin concentration [mIU/mL]/22.5.

The statistical analysis was performed using MedCalc 10.2.0.0 software. Nonsequitur was assumed at 5%. The findings, whose value was equal to *p* < 0.05, were considered statistically significant. The quantitative variables such as the concentration of the tested parameters (IGF-1, IGF-2, IGFBP-1, IGFBP-2, IGFBP-3, insulin, glucose) and certain demographic and clinical factors (e.g., age, body weight, BMI) were analyzed by descriptive statistics: measurement of concentration (median, mean, maximum value and minimum value) and measures of dispersion (standard deviation). Using the U–Mann–Whitney test (variables where the distribution is different from normal) or Student’s t-test (variables with normal distribution), the authors compared the distribution of values of the examined parameters (IGF-1, IGF-2, IGFBP-1, IGFBP-2, IGFBP-3, insulin, glucose) in the examined group and in the control group, and assessed a relationship with selected demografic-clinical factors in the test group. By means of Spearman’s test, the correlation between the examined parameters (IGF-1, IGF-2, IGFBP-1, IGFBP-2 and IGFBP-3) and the selected clinical factors was assessed.

## 3. Results

**Figure 1:**
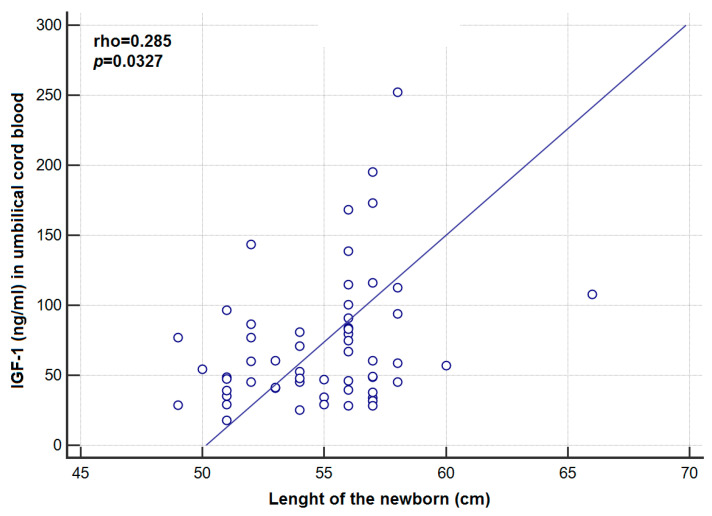
Correlation between concentration of IGF-1 in umbilical cord blood and the length of the newborn in the GDM group.

Table 1 presents a comparison of the study group (GDM) and the control group (C). The analysis of the concentrations of both IGF-1, -2 and IGFBP-3 in peripheral blood and umbilical cord blood showed no statistically significant difference between the GDM group and the Cgroup (Table 2). A significantly lower concentration of IGFBP-1 in peripheral blood (*p* = 0.0001) and umbilical cord blood (*p* = 0.0443) were shown in the GDM group compared to the C group. The above, statistically significant differences were confirmed only for patients with BMI < 25 (*p* = 0.0003 and *p* = 0.0476, respectively). A significantly lower concentration of IGFBP-2 in peripheral blood (*p* = 0.0217) and umbilical cord blood (*p* = 0.0001) was shown in the GDM group compared to the C group. The above relationships were confirmed only for patients with BMI < 25 (*p* = 0.0217 and *p* = 0.0001, respectively). A statistically significantly higher level of insulin (*p* = 0.0210) in the peripheral blood of women with GDM compared to healthy women was demonstrated (the above relationship was not confirmed in BMI subgroups); the analysis of the concentration of peripheral blood glucose in women with GDM and in healthy women showed no significant difference (*p* = 0.1117). There was also observed a significantly higher median of the indicator value HOMA-IR in the GDM group compared to the C group (5.69 vs. 4.37; *p* = 0.0150). However, this relationship was confirmed only in BMI > 24.99 (6.37 vs. 3.40; *p* = 0.0017) (Table 2). A significantly higher level of IGF-1 (*p* = 0.0436) was found in the cord blood of newborns delivered by women suffering from GDM, who had been on a diet vs. insulin. Correlations between maternal and cord plasma IGF-1, IGF-2, IGFBP-1-3 concentrations and clinical/biochemical parameters in the GDM group are presented in Table 3 and Table 4. A statistically significant positive correlation was observed between the weight gain during pregnancy and the concentration of IGF-2 in the peripheral blood of women with GDM (rho = 0.263; *p* = 0.0433), as well as between the concentration of IGF-1 (ng/mL) in the serum of umbilical cord blood of newborns delivered by women with GDM, and the newborn length soon after birth (rho = 0.285; *p* = 0.0327) (Figure 1). A statistically significant positive correlation was observed between the concentration of IGF-1 and IGF-2 in the peripheral blood of women with GDM and the IGF-1 concentration (rho = 0.352; *p* = 0.0069), and the concentration of IGF-2 (rho = 0.644; *p* < 0.0001) in the serum of umbilical cord blood of newborns delivered by women with GDM. In the study group, the drop in IGFBP-2 concentration in maternal blood was accompanied by an increase of IGF-2 in newborn’s cord blood (rho = −0.367, *p* = 0.0049). There was a statistically significant negative correlation between the value of the indicator HOMA-IR, and the concentration of IGFBP-2 in the umbilical cord blood of newborns delivered by healthy women (rho = −0.28; *p* = 0.0487). However, in the GDM group, such a relationship was not found. Comparisons of different IGF concentrations according to weight classification based on BMI were performed with the use of ANOVA Kruskal–Wallis test. There were no statistically significant differences in the concentrations of the tested laboratory markers depending on adult body weight classification based on BMI. Only in the case of IGFBP-1, some trend towards significantly higher values of this marker was noted in women with GDM and BMI < 25 (with desirable body weight) compared to the subjects with BMI > 24.99 (overweight) and BMI > 29.99 (obese) (118.06 vs. respectively: 72.77 and 62.40; *p* = 0.0548) (Table 5).

## 4. Discussion

The data in the accessible literature on the role of insulin-like growth factors for gestational diabetes are inconsistent. Most of the studies assessed IGF parameters either in fetal or maternal circulation, while in this study, we decided to evaluate the IGF system in both compartments [6,7]. Conflicting results are also provided by a study evaluating the effect of IGF on the process of the growth of the fetus in physiological pregnancy and in GDM [8,9,10]. 

### 4.1. Assessment of IGF-1 Concentration in the Serum of Maternal Blood

IGF-1 circulating in the peripheral blood is mainly produced by liver cells and endothelial cells. It contributes to the process of glucose transport to insulin sensitive tissues via specific receptors as well as insulin receptors, which exert a hypoglycemic effect. Studies revealed that IGF-1 suppresses hepatic glucose production [11,12]. Due to such a profile of action, its role in pregnancy complicated by diabetes seems to be of utter importance. Yet, the subject literature provides conflicting data with regard to the concentration of IGF-1 in peripheral blood in patients with GDM. Sesti et al. found decreased levels of IGF-1 concentration in the blood serum of patients suffering from diabetes [10]. However, in her report, Lappas did not establish any significant difference between the concentration of IGF-1 in maternal serum in the course of physiological pregnancy and in GDM pregnancy [13]. In the investigated clinical material, the analysis of IGF-1 concentration in peripheral blood of women with GDM and in healthy patients did not reveal a statistically significant difference, either. Similar results were obtained by other authors [14,15]. It can be assumed that, due to a significant improvement in the quality of care over pregnant women with gestational diabetes that has occurred over the last several years, the disorders observed by Sesti et al. were not confirmed in subsequent reports. However, a direct impact of serum, maternal IGF-1 concentrations on the process of fetal growth seems to be unlikely [13,15]. The circulating IGF-1 in the maternal bloodstream does not exceed the placental barrier, which might be suggested by its higher concentration in the mother’s blood serum compared to the concentration in fetal blood serum [15]. In the author’s own study, such a difference was also reported.

### 4.2. Assessment of the Concentration of IGF-1 in the Serum of Umbilical Cord Blood

Fetal IGF-1, with its mitogenic and metabolic actions, is believed to be an important mediator in fetal growth [16]. Schwartz et al. reported that the increase in the concentration of IGF-1 in the umbilical cord blood is responsible for accelerating the intrauterine growth of the fetus, and the decrease in the serum concentration of IGFBP-1 in patients with GDM increases the bioavailability of IGF-1, which leads to the development of macrosomia [17]. This theory was confirmed by the study of Luo et al., who found a positive correlation between the concentration of insulin and IGF-1 in the fetus and birth weight of a newborn [18]. Moreover, Lindsay et al. achieved comparable concentration values with the author in a group of newborns delivered by mothers with GDM—42.2 ± 16.3 ng/mL and 34.7 ± 18.5 ng/mL in the control group [19]. Nonetheless, the obtained results in own research did not reach the level of statistical significance in contrast to Lindsay et al., which proved significantly higher levels of IGF-1 in the serum of umbilical cord blood in a group of newborns delivered by mothers with GDM as compared to the control group [19]. The above results were confirmed a few years later in another report [18]. It has been demonstrated that a significant effect on the secretion of IGF-1 in the fetus is exerted by insulin [20]. The results obtained by the quoted researchers can also be explained by an increased supply with glucose for the fetus in case of GDM, a secondary increase in insulin secretion in the fetus and an increase in fetal IGF-1 concentration in the umbilical cord. Surprising results were published by Wang et al. recently. They found elevated cord blood IGF-I level in GDM pregnancies, without a significant difference in cord blood insulin concentrations between the newborns of GDM pregnancies and controls [6]. This may be related to the fact that cord blood insulin levels might only reflect very short-term glycemic status when pregnant patient were euglycemic due to proper treatment. In contrast to the above studies, the results consistent with the findings achieved in the author’s own studies were also proven in other reports [13,21]. The discrepancies in the results of the studies cited above may result from a varying degree of GDM control in the examined patients. It is worth mentioning that, in the study made by Lindsay et al., the birth weight of newborns delivered by mothers with GDM was statistically higher than in the control group, which may indirectly suggest lack of optimal control of GDM [19]. Such a difference in terms of birth weight in newborns was not observed in own research, where the newborns weight in the control and study group was similar. It can therefore be assumed that the optimal control of GDM in the examined patients did not affect the concentration of IGF-1 in the blood of the umbilical cord in the discussed group of patients.

### 4.3. Assessment of IGF-2 Concentration in the Serum of Maternal Blood

There was no statistically significant difference between the concentration of IGF-2 in maternal blood serum during the physiological pregnancy and pregnancy with GDM [13]. In the self-study, similar results were obtained, since the analysis of IGF-2 concentration in the patients’ peripheral blood, both in the examined group and in the control group, showed no statistically significant difference, which is also consistent with the observations made by Luo et al. [18]. The research results suggest that GDM does not have a significant effect on IGF-2. In the author’s own research, there was no correlation between the concentration of IGF-2 in the blood serum of the mother and the newborn birth weight, which is confirmed in the reports made by Luo et al. [18]. It can therefore be concluded that IGF-2 has no effect on the birth weight of the newborn. Positive correlations between maternal IGF-2 concentration and weight gain during pregnancy in the study group could be caused by increased secretion from adipose tissue in response to hyperglycemia.

### 4.4. Assessment of the Concentration of IGF-2 in the Serum of Umbilical Cord Blood

In the study by Luo et al., there were no statistically significant differences between the level of IGF-2 in the serum of cord blood in patients with GDM and in healthy women [18]. The effect of GDM on the level of IGF-2 in a newborn was not confirmed by Lappas either [13]. In the above-mentioned study, there was no difference in IGF-2 concentration in the maternal and fetal blood between SGA (small for gestational age), AGA (appropriate for gestational age) and LGA (large for gestational age) newborns. The above observations were also confirmed by the results of the author’s own study, in which there was no statistically significant difference between IGF-2 concentration of umbilical cord blood in newborns delivered by women with GDM and by healthy mothers. The lack of increased IGF-2 concentration in umbilical cord blood, with regard to GDM, may be dependent on insulin secretion of IGF-2 in a fetus (as opposed to IGF-1). Some researchers believe, however, that IGF-2 plays a major role, both in the process of growth and in the embryonic period [22]. On the other hand, in the fetal stage, the dominant role in this process is attributed to IGF-1 [23]. In the Wang et al. study, an elevated level of umbilical cord IGF-2 in GDM patients was observed, which may explain its influence on fetal growth [6]. This issue requires further research. A statistically significant positive correlation between the value of the indicator HOMA-IR and the concentration of IGF-2 in umbilical cord blood of newborns delivered by women suffering from GDM may suggest a potential role of IGF-2 in the development of insulin resistance in utero.

### 4.5. Assessment of IGFBP-1 Concentration in the Serum of Maternal Blood

The study provided by Lappas demonstrated that the concentration of IGFBP-1 in maternal blood was statistically lower compared to pregnant women without disorders of carbohydrate metabolism [13]. Similar results were obtained in the author’s own research. In the comparative analysis of IGFBP-1 concentration in the peripheral blood of patients with GDM and in healthy patients, a significantly lower IGFBP-1 concentration in the first group was demonstrated. IGFBP-1 may also play a role in glucose metabolism through its impact on free IGF-1 level. [24] The fall in IGFBP-1 concentration in GDM may cause an increase in free IGF-1 in a pregnant woman, which, in turn, is connected with increased placenta weight, and consequently, a better supply of the fetus with nutrients. It seems that the mechanism mentioned above can account for accelerated fetal growth in the case of GDM. However, in our study, no statistically significant relationship was found between maternal IGFBP-1 concentration and newborn weight, which may be associated with the optimal control of diabetes in this group of patients. 

### 4.6. Assessment of the Concentration of IGFBP-1 in the Serum of Umbilical Cord Blood

In the report made by Lappas in 2015, it was proven that the concentration of IGFBP-1 in umbilical cord blood, similarly to maternal blood, was significantly lower in newborns delivered by mothers with GDM, compared to babies without GDM [13]. Lower IGFBP-1 concentration in umbilical blood was also found in babies born by patients with GDM, unlike those born by mothers with correct carbohydrate tolerance [19]. Findings which are similar to those presented above were obtained in the author’s own study, in which lower IGFBP-1 was demonstrated in the umbilical cord blood of babies born to women with GDM, as compared to babies born to healthy mothers. Different results were provided in a study by Kanai et al., who did not observe a statistically significant difference between IGFBP-1 concentration in umbilical cord blood of babies born to healthy mothers and those with GDM [21].

The results, obtained in this work, also confirmed by other researchers, with regard to decreased IGFBP-1 in babies born to mothers with GDM, can be explained by hyperglycemia and an increased insulin concentration in the fetus, which inhibits the production of IGFBP-1 in the liver.

### 4.7. Assessment of IGFBP-2 Concentration in the Serum of Maternal Blood

The results of the author’s own research point to a statistically significantly lower IGFBP-2 concentration in the peripheral blood serum of women with GDM, compared to the concentration in the blood serum of women with a physiological course of pregnancy. The obtained results suggest that GDM has an effect also on IGFBP-2 in maternal blood serum. In a longitudinal study in a multiracial cohort, concentrations of IGFBP-2 were significantly lower in 107 case subjects with GDM than control subjects at gestational weeks 10–14 and 15–26 [5]. These findings are in agreement with a previous study performed on nonpregnat women with type 2 diabetes [25]. At the cellular level, IGFBP-2 may inhibit IGFs by competing with their receptors for peptide binding and regulate the IGF axis [26]. IGF-independent actions of IGFBP-2 are well known. It can bind to α_5_β_1_-integrin receptors and activate a downstream signaling cascade via the phosphatidylinositol-3-kinase and protein kinase β pathway, which is implicated in glucose uptake and insulin sensitivity [27]. The pleiotropic actions of IGFBP-2 give it a crucial role in metabolic regulation and glucose homeostasis.

### 4.8. Assessment of the Concentration of IGFBP-2 in the Serum of Umbilical Cord Blood

In Kanai et al.’s study, the average concentration of IGFBP-2 in umbilical cord blood in a group of pregnant women without glucose tolerance equaled 926 ± 364 ng/mL and was comparable with the concentration of this protein in the group with GDM—965 ± 467 ng/mL [21]. The results of the author’s own research indicate a statistically significantly lower IGFBP-2 concentration in the umbilical cord blood serum of babies born to women with GDM, as compared to the concentration of IGFBP-2 in the blood serum of healthy ones. The median concentration of IGFBP-2 in the peripheral blood of babies born to women with GDM was equal to 796.10 ng/mL On the other hand, the median concentration of IGFBP-2 in the umbilical cord blood of babies born to healthy women was 1101.80 ng/mL. Similar results were obtained by Lappas [13]. The decrease in the concentration of IGFBP-2 leads to an increase of biologically active insulin-like growth factors that may accelerate the growth of the fetus in the case of GDM.

### 4.9. Assessment of IGFBP-3 Concentration in the Serum of Maternal Blood

In the investigated clinical material, the analysis of IGFBP-3 concentration in the blood of women with GDM and in healthy patients did not prove to be a significant statistically difference. The findings are consistent with different reports [5,13]. Meanwhile, in Grissa et al.’s study, IGFBP-3 concentrations in late gestation were significantly higher in GDM vs. euglycemic pregnancies [28]. Wang and co-workers’ review revealed that IGFBP-3 concentrations do not change in the first trimester until in the late pregnancy [4]. This may suggest that observed changes appear secondary to GDM and it seems that they should not be taken as a cause of disease.

### 4.10. Assessment of the Concentration of IGFBP-3 in the Serum of Umbilical Cord Blood

Previous studies on concentrations of IGFBP-3 in the umbilical cord blood serum of newborns delivered by mothers with GDM remain inconsistent. Schwartz et al. report that IGFBP-3 concentration increases in pregnancy complicated with diabetes [17]. Yan-Jun et al. also observed increased concentrations of IGFBP-3 in newborns delivered by mothers whose pregnancies were complicated by diabetes [29]. However, different results were provided by the Lappas investigation, in which there was a statistically lower concentration of IGFBP-3 in umbilical cord blood in newborns delivered by women with GDM, compared to newborns delivered by non-GDM mothers [13]. In the authors’ own study, when comparing the concentrations of IGFBP-3 in the umbilical cord blood between the study and control group, no statistically significant difference was found. Due to the divergent results, further research seems necessary.

The summary of the studies on IGF system in GDM and euglycemic control group in late pregnancy conducted so far is presented in the Table 6.

There were some limitations to this study. First, it was performed using only one sample of blood at the beginning of the first stage of labor. In the future, it would be advisable to perform serial determinations in the three trimesters of pregnancy, and after delivery. Secondly, it would be worth making such an analysis on a larger group of patients.

## 5. Conclusions

GDM does not significantly affect the concentrations of IGF-1, -2 and IGFBP-3 in the peripheral blood and umbilical cord blood. A positive correlation between the concentration of IGF-1 in umbilical cord blood and the length of the newborn body indicates the influence of IGF-1 upon the process of the fetus growth in the case of GDM. Collectively, our findings suggest that GDM has a greatest influence on maternal and fetal IGFBP-2 concentrations. Understanding the specific role played by the insulin-like system of growth factors in the development of GDM and its complications may, in the future, enable appropriate preventive measures. Further research which evaluates the auto- and paracrine role of the IGF system is essential.

## Figures and Tables

**Table 1:** Characteristics of the study group (GDM) and the control group (C)—Comparison of selected parameters.

Factor	GDM Group (*n* = 60)	C Group (*n* = 49)	*p*
	M ± SD ^a^ or *Me* (95%CI) ^b^		
Age (years)	31.02 ± 5.05 ^a^	29.00 (28.00–31.00) ^b^	0.0857
Gestational age (week)	38.6 ± 1.58 ^b^	40 (39–40) ^a^	0.0012
Body weight before pregnancy (kg)	70.13 ± 13.20 ^a^	61.00 (59.17–64.00) ^b^	0.0017
BMI at present (kg/m^2^)	30.43 ± 4.88 ^a^	26.29 (25.37–28.36) ^b^	0.0003
BMI before pregnancy (kg/m^2^)	25.75 ± 4.46 ^a^	22.04 (21.35–23.42) ^b^	<0.0001
Weight gain (kg)	12.78 ± 5.53 ^a^	13.12 (10.00–15.00) ^b^	0.7599
Newborn’s weight (g)	3459.66 ± 601.24 ^a^	3377.04 ± 425.66 ^a^	0.4197
Newborn’s length (cm)	55.50 (54.00–56.00) ^b^	53.41 ± 2.67 ^a^	0.0554

^a^ results for variables with normal distribution have been presented as mean (M) ±SD (standard deviation) ^b^ results for variables with a distribution different from the normal one have been presented as *Me* (median) and its 95% CI (confidence interval). Abbreviations: GDM—Gestational Diabetes Mellitus, C—control, BMI—Body Mass Index.

**Table 2:** A comparison of the examined parameters in the peripheral blood of both women with GDM and healthy women (C), as well as in umbilical cord blood of newborns delivered by women with GDM and by healthy mothers (C).

Factor	C	GDM	*p*	C	GDM	*p*
	Maternal Blood			Cord Blood		
	*Me* (95%CI)			*Me* (95%CI)		
**IGF-1 (ng/mL)**	213.37 (184.86–258.55)	262.98 (218.71–292.42)	0.0791	48.10 (43.15–55.37)	55.52 (46.83–75.06)	0.1044
BMI > 24.99	185.89 (145.26–310.21)	249.27 (209.32–281.91)	0.1474	53.29 (32.62–75.55)	48.35 (40.93–58.65)	0.9749
BMI < 25	213.37 (179.45–289.31)	289.31 (219.43–319.57)	0.1907	48.1 (43.10–55.54)	73.87 (46.62–92.96)	0.0226
**IGF-2 (ng/mL)**	756.81 (698.06–802.24)	729.22 (700.28–800.58)	0.5071	387.7450 (354.20–426.95)	360.48 (311.53–387.45)	0.0544
BMI > 24.99	861.39 (721.43–1020.04)	791.34 (688.41–852.49)	0.2914	382.95 (301.21–503.54)	375.85 (321.15–409.72)	0.4310
BMI < 25	739.30 (686.93–772.01)	719.64 (671.04–773.90)	0.5010	672.39 (353.33–423.34)	311.91 (253.98–396.83)	0.0634
**IGFBP-1 (ng/mL)**	155.01 (95.92–182.25)	69.56 (49.57–107.05)	0.0001	53.31 (47.52–62.45)	34.91 (29.23–50.14)	0.0443
BMI > 24.99	78.17 (42.01–193.43)	66.38 (48.93–120.28)	0.7528	49.42 (16.55–77.73)	35.74 (27.65–64.73)	0.8501
BMI < 25	162.04 (126.71–195.36)	75.56 (47.60–111.47)	0.0003	53.31 (47.37–63.03)	33.98 (23.70–53.62)	0.0476
**IGFBP-2 (ng/mL)**	116.97 (104.49–137.27)	107.24 (93.03–116.17)	0.0217	1101.80 (968.59–1266.56)	796.10 (694.90–902.00)	0.0001
BMI > 24.99	101.83 (81.83–188.45)	110.17 (89.21–130.28)	0.9623	1280.46 (778.14–1343.26)	907.53 (744.99–981.51)	0.0588
BMI < 25	117.93 (105.94–148.24)	104.27 (81.72–121.97)	0.0219	1064.83 (933.13–1210.58)	697.05 (542.09–841.65)	0.0001
**IGFBP-3 (ng/mL)**	4607.34 (4471.44–4870.80-)	5191.11 (4749.07–5542.05)	0.0708	1250.40 (1171.08–1355.47	1279.18 (1175.91–1373.50)	0.9593
BMI > 24.99	4700.28 (4131.66–4899.61)	5138 (4678.64–5866.62)	0.1388	1239.31 (1100.66–1380.34)	1212.79 (1102.67–1355.58)	0.9749
BMI < 25	4607.34 (4426.25–5120.80)	5315.94 (4557.85–5815.31)	0.2082	1245.89 (1164.44–1405.67)	1333 (193.64–1593.39)	0.4584
**Insulin (mIU/mL)**	26.46 (20.31–32.40)	33.90 (28.17–37.46)	0.0210	−	−	−
BMI > 24.99	24.29 (17.13–35.53)	33.90 (27.24–51.91)	0.1388	−	−	−
BMI < 25	27.52 (20.31–35.30)	33.81 (23.11–37.58)	0.2811	−	−	−
**Glucose (mmol/L)**	3.70 (3.67–3.79)	3.80 (3.67–3.89)	0.1117	−	−	−
BMI > 24.99	3.59 (3.43–3.96)	3.86 (3.66–4.12)	0.1706	−	−	−
BMI < 25	3.72 (3.67–3.80)	3.76 (3.62–3.89)	0.5920	−	−	−
**HOMA-IR**	4.37 (3.35–5.31)	5.69 (4.38–6.76)	0.0150	−	−	−
BMI > 24.99	3.40 (2.90–4.97)	6.37 (4.28–8.57)	0.0017	−	−	−
BMI < 25	5.23 (3.55–8.19)	5.33 (3.34–6.20)	0.9766	−	−	−

Abbreviations: GDM—Gestational Diabetes Mellitus, C—control, *Me*—median, IGF—insulin-like growth factor, IGFBP—Insulin-like growth factor-binding protein, HOMA-IR—homeostasis model assessment of insulin resistance, BMI—Body Mass Index.

**Table 3:** Spearman’s correlation between maternal plasma (IGF-1, IGF-2, IGFBP-1, IGFBP-2, IGFBP-3) concentrations (ng/mL) and clinical/biochemical parameters in the GDM group.

Variable	Rho (*p*)				
	IGF-1	IGF-2	IGFBP-1	IGFBP-2	IGFBP-3
**APGAR**	0.135	0.001	−0.149	−0.088	0.039
	(0.3120)	(0.9921)	(0.2652)	(0.5096)	(0.7663)
**Birth weight of the newborn [g]**	0.023	0.132	0.124	−0.074	−0.024
	(0.8620)	(0.3239)	(0.3537)	(0.5772)	(0.8547)
**Birth length of the newborn [cm]**	0.159	0.047	−0.064	−0.064	0.084
	(0.2353)	(0.7209)	(0.6319)	(0.6305)	(0.5278)
**OGTT (fasting) [mg/dl]**	−0.089	0.030	−0.111	−0.145	−0.078
	(0.5519)	(0.8393)	(0.4599)	(0.3359)	(0.6008)
**OGTT (75 g 2 h) [mg/dl]**	0.199	−0.005	−0.228	−0.033	0.086
	(0.1861)	(0.9730)	(0.1304)	(0.8245)	(0.5653)
**Weight (current) [kg]**	−0.0173	0.188	−0.0465	0.0183	0.00427
	(0.8944)	(0.1483)	(0.7210)	(0.8879)	(0.9739)
**Weight (initial) [kg]**	−0.0517	0.153	−0.0344	0.00742	−0.0232
	(0.6914)	(0.2398)	(0.7918)	(0.9545)	(0.8586)
**Weight gain during pregnancy [kg]**	−0.0254	0.263	0.0104	0.0469	0.0109
	(0.8453)	(0.0433)	(0.9364)	(0.7187)	(0.9336)
**BMI (before pregnancy) [kg/m^2^]**	−0.0673	0.194	0.00661	0.0622	−0.00122
	(0.6052)	(0.1364)	(0.9595)	(0.6328)	(0.9925)
**HOMA-IR**	0.122	−0.153	−0.116	−0.129	0.066
	(0.3678)	(0.2574)	(0.3910)	(0.3372)	(0.6234)

Abbreviations: APGAR—Activity, Pulse, Grimace, Appearance and Respiration, BMI—body mass index; HOMA-IR—homeostasis model assessment-insulin resistance; IGF—insulin growth factor, IGFBP—insulin growth factor-binding protein; OGTT—oral glucose tolerance test.

**Table 4:** Spearman’s correlation between cord blood plasma (IGF-1, IGF-2, IGFBP-1, IGFBP-2, IGFBP-3) concentrations (ng/mL) and clinical/biochemical parameters in the GDM group.

Variable	Rho (*p*)				
	IGF-1	IGF-2	IGFBP-1	IGFBP-2	IGFBP-3
**APGAR**	0.027	0.052	0.022	−0.104	−0.044
	(0.8391)	(0.6972)	(0.8654)	(0.4348)	(0.7418)
**Birth weight of the newborn [g]**	0.128	0.159	−0.017	−0.080	0.043
	(0.3367)	(0.2344)	(0.8946)	(0.5492)	(0.7425)
**Birth length of the newborn [cm]**	0.285	0.028	−0.084	−0.088	0.199
	(0.0327)	(0.8313)	(0.5273)	(0.5103)	(0.1355)
**OGTT (fasting) [mg/dl]**	0.056	0.009	0.142	−0.136	−0.166
	(0.7080)	(0.9513)	(0.3473)	(0.3660)	(0.2704)
**OGTT (75 g 2 h) [mg/dl]**	0.128	0.002	−0.211	0.026	−0.076
	(0.3960)	(0.9894)	(0.1613)	(0.8618)	(0.6101)
**Weight (current) [kg]**	−0.152	0.145	−0.0415	0.0198	−0.109
	(0.2429)	(0.2652)	(0.7497)	(0.8792)	(0.4031)
**Weight (initial) [kg]**	−0.218	0.114	−0.0218	0.0893	−0.128
	(0.0937)	(0.3806)	(0.8670)	(0.4926)	(0.3240)
**Weight gain during pregnancy [kg]**	−0.183	0.199	−0.0483	0.0882	−0.141
	(0.1606)	(0.1266)	(0.7105)	(0.4983)	(0.2804)
**BMI (before pregnancy) [kg/m^2^]**	−0.1953	0.147	−0.0327	0.167	−0.146
	(0.1348)	(0.2577)	(0.8017)	(0.1990)	(0.2632)
**HOMA-IR**	0.033	−0.101	0.212	−0.121	0.115
	(0.8083)	(0.4550)	(0.1134)	(0.3686)	(0.3937)

Abbreviations: APGAR—Activity, Pulse, Grimace, Appearance and Respiration, BMI—body mass index; HOMA-IR—homeostasis model assessment-insulin resistance; IGF—insulin growth factor, IGFBP—insulin growth factor-binding protein; OGTT—oral glucose tolerance test.

**Table 5:** Comparisons of studied laboratory markers according to adult GDM body weight classification based on BMI.

Variable	BMI			*p*
Maternal Blood	<25	25–29.99	>29.99	
IGF-1	248.78 (167.25–336.58)	238.79 (184.80–320.86)	245.91 (176.05–339.97)	0.9962
IGF-2	726.58 (657.85–819.59)	801.25 (618.20–890.13)	805.55 (582.52–918.38)	0.2556
IGFBP-1	118.06 (62.96–194.08)	72.77 (46.64–132.42)	62.40 (25.25–207.17)	0.0548
IGFBP-2	112.13 (90.23–147.36)	106.26 (87.17–145.06)	93.28 (73.65–131.99)	0.4614
IGFBP-3	4745.70 (4273.07–5879.77)	4998.14 (4247.76–5951.03)	4829.96 (4578.91–5643.28)	0.9816

Abbreviations: GDM—Gestational Diabetes Mellitus, IGF—insulin-like growth factor, IGFBP—Insulin-like growth factor-binding protein, BMI—Body Mass Index.

**Table 6:** Summary data in studies on IGF-1, IGF-2 and IGFBP-1-3 levels in GDM and control (euglycemic) pregnancies.

Factor	References	Study Type *	Country	Gestational Age [Weeks]	GDM (ng/mL)			Control (ng/mL)			GDM vs. Control Difference (95% Cl) ^a^
					N	Mean	SD	N	Mean	SD	
IGF-1	Luo et al. [18]	PC	Canada	32–35	27	403.6	171.8	279	307.0	123.6	**−96.60 (30.19, 163.01)**
	Zhu et al. [5]	NCC	USA	37–39	51	326.5	302.0	58	297.1	216.3	29.41 (70.43, 129.25)
	Grissa et al. [28]	PC	Tunisia	Delivery	30	650.9	168.0	30	414.3	100.6	**236.66 (166.59, 306.73)**
	Lappas [13]	CC	Australia	Delivery	44	58.0	35.8	30	57.4	25.7	0.60 (−13.42, 14.62)
IGF-2	Luo et al. [18]	PC	Canada	32–35	27	983.5	244.8	279	995.7	180.4	−12.20 (−106.93, 82.53)
	Lappas [13]	CC	Austarlia	Delivery	44	293.7	189.0	30	269.5	175.8	24.20 (−67.43, 115.83)
IGFBP-1	Lappas [13]	CC	Australia	Delivery	44	54.4	33.8	30	72.3	41.1	−17.90 (−35.77, −0.03)
IGFBP-2	Lappas [13]	CC	Australia	Delivery	44	157.8	109.4	30	189.30	126.5	−31.50 (−87.12, 24.12)
	Zhu et al [5]	NCC	USA	36–39	51	87.44	15.53	58	91.87	15.53	−4.43 (−10.27, 1.41)
IGFBP-3	Zhu et al [5]	NCC	USA	36–39	51	5085.7	1602.9	58	4965.7	1922.5	120.0 (−542.1, 782.1)
	Grissa et al. [28]	PC	Tunisia	Delivery	30	1836.5	912.9	30	1302.6	912.8	**533.9 (71.9, 995.9)**
	Lappas [13]	CC	Australia	Delivery	44	3997.4	996.3	30	3693.7	1081.2	303.7 (−182.5, 789.9)

* Study type: PC-prospective cohort study; NCC-nested case–control study; CC-case–control study; ^a^ The differences with 95% CIs excluding the zero are shown in bold.
